# The planar cell polarity protein Vangl2 interacts with the PDZ‐domains of Scribble but not with a unique PDZ‐like domain in Inturned

**DOI:** 10.1002/1873-3468.70319

**Published:** 2026-03-08

**Authors:** Stephan Wilmes, Jan Brysch, Carmen Gelze, Lilli Meier, Daniel Kümmel

**Affiliations:** ^1^ Department of Chemistry and Pharmacy, Institute of Biochemistry University of Münster Germany

**Keywords:** Intu, PDZ domain, protein–protein interaction, Scrib, Vang, X‐ray crystallography

## Abstract

The proteins Inturned and Fuzzy are members of the tri‐longin domain (TLD) RabGEF family and activate the GTPase Rab23 downstream of the core planar cell polarity (PCP) proteins Vangl2 and Prickle. To gain insight into the function of a predicted PDZ domain unique to Inturned among TLD proteins, we performed structural and biochemical characterisations. We show that this domain does not interact with membranes or Vangl2. Instead, we find a phosphorylation‐dependent interaction between Vangl2 and a PDZ domain of the apical‐basal polarity protein Scribble. A crystal structure of Intu‐PDZ reveals a unique PDZ‐like fold lacking an interaction site for PDZ‐binding motifs. Our data provide new insight into the role of PDZ domains in coordinating cell polarity downstream of Vangl2.

## Graphical abstract

Structural and biochemical characterisations show that the planar cell polarity (PCP) protein Inturned harbours a unique PDZ‐like domain that does not bind canonical PDZ‐binding motifs (PBMs) like that of another PCP protein Vangl2. In contrast, the apical‐basal polarity protein Scribble contains four PDZ domains that bind Vangl2, but one PDZ domain requires Vangl‐PBM phosphorylation for stable interaction.

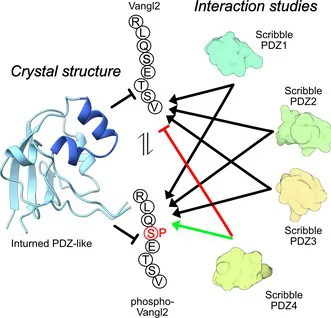

## Abbreviations

**BLI**, biolayer interferometry

**CPLANE**, ciliogenesis and planar polarity effector

**DTT**, dithiothreitol

**GAP**, GTPase activating protein

**GDP**, guanosine diphosphate

**GEF**, guanine nucleotide exchange factor

**GST**, glutathione S‐transferase

**GTP**, guanosine triphosphate

**ipTM**, interface predicted template modelling

**PBM**, PDZ binding motif

**PCP**, planar cell polarity

**PDZ**, Postsynaptic density protein 95, Discs large, Zonula occludens‐1

**PI3P**, phosphatidylinositol 3‐phosphate

**Pk**, Prickle

**PPE**, planar polarity effector

**pSer517**, Vangl‐2 (peptide) with phosphorylation at serine 517

**pTM**, predicted template modelling

**PVDF**, polyvinylidene fluoride

**Scrib**, Scribble

**TLD**, tri‐longin domain RabGEF

**TCEP**, tris(2‐carboxyethyl)phosphin

**Vang(l)**, Van Gogh(−like)

**wt**, wild‐type

Planar cell polarity (PCP) is a fundamental morphogenetic process that orchestrates the orientation of cells along the anterior‐to‐posterior tissue axis [1]. Among other proteins, PCP requires Fuzzy and Inturned (Intu), which together act as guanine nucleotide exchange factor (GEF) for the GTPase Rab23 [2, 3]. Intu and Fuzzy have been shown to form a trimeric complex with Fritz in flies called planar polarity effector (PPE) [4]. In mammalian cells, Intu and Fuzy form the CPLANE (ciliogenesis and planar polarity effector) complex together with WDPCP (vertebrate homologue of Fritz), the atypical small GTPase Rsg1 (CPLANE‐2) and the poorly characterised protein JBTS17 (CPLANE‐1) [4, 5]. CLPANE participates in ciliogenesis and localises to the basal body of the primary cilium where it is needed for axoneme elongation, fusion of the ciliary vesicle to the plasma membrane [2], and recruitment of IFT‐A proteins that are required for intraciliary trafficking [5].

On a molecular level, the GEF activity of Fuzzy‐Inturned mediates the switch of Rab23 from the inactive guanosine diphosphate (GDP)‐bound to the active guanosine triphosphate (GTP)‐bound state [3]. Rab GTPases are identity markers for organelle identity and sites of membrane fusion, and spatiotemporal control of their activation critically depends on regulation by their cognate GEFs [6].

Fuzzy‐Inturned belong to the family of heterodimeric tri‐longin domain (TLD) RabGEFs, which also includes Hps1‐Hps4 (also known as BLOC‐3) that controls biogenesis of lysosome‐related organelles [7] and Mon1‐Ccz1 that regulates endosomal maturation and autophagy [8, 9]. TLD RabGEFs use a conserved catalytic mechanism [10, 11, 12] but differ in substrate specificity and cellular localisation. Unique subunits and domains are thought to direct the GEF complexes to their respective target compartments. Mon1‐Ccz1 is the best studied TLD RabGEF and was shown to rely on the synergistic effects of lipid‐protein and protein–protein interactions with different recruiter proteins [13, 14]. Recently, colocalisation of Rsg1 and the Bin/Amphiphysin/Rvs (BAR) domain containing protein 1 (CiBAR1, also known as FAM92A1) to the ciliary base [15, 16] and their interaction with Fuzzy‐Inturned were shown [11, 17], which likely represents the recruiting mechanism of CPLANE in ciliogenesis. How Fuzzy‐Inturned is recruited to membranes in planar cell polarity remains unclear.

Intu contains a predicted PDZ (Postsynaptic density protein 95/Discs large/Zonula occludens‐1) domain (Intu‐PDZ) as a unique feature among TLD family proteins [18]. Due to flexibility, this domain was not resolved in previous Fuzzy‐Inturned complex structures determined by cryo‐EM [11, 19]. PDZ domains are widely spread and are known to facilitate predominantly protein–protein interactions [20, 21]. They have a distinct, highly conserved fold with a five‐ or six‐stranded β‐sheet sandwiched by two α‐helices. Interacting PDZ binding motifs (PBM) bind to a groove between α2 and β2 [22]. Commonly, but not exclusively, the PBM is a C‐terminal sequence, which binds an R/K‐XXX‐GLGF motif in its target PDZ domain via hydrogen bonds with its terminal carboxylate. Intu‐PDZ was previously reported to interact with the transcription factor Stat1 based on colocalisation and co‐immunoprecipitation experiments, suggesting a role of Intu in proteasomal degradation of Stat1 [23]. However, a clear molecular mechanism for these findings remains elusive.

The transmembrane protein Van Gogh (Vang, also known as Strabismus) in flies and its vertebrate orthologues Vangl1 and Vangl2 (Vang Gogh‐like) asymmetrically localise to the proximal side of cells. Together with the membrane protein Flamingo (in flies)/ Celsr (in vertebrates) and cytosolic Prickle, they are essential for establishing PCP [24]. Recent structural studies showed that Vangl proteins form trimers [25, 26]. Interestingly, Vang and Vangl contain a conserved PBM and the PDZ domain proteins Sorting Nexin 27 [27], Gipc1 [28], MAGI‐3 [29] and Scribble (Scrib) [30] represent Vangl interactors. Scribble is involved in apical‐basal polarity and regulation of cell proliferation [31, 32, 33] and contains four PDZ domains. All Scrib PDZ domains have been reported to interact with Vangl‐PBM [34, 35, 36] although results varied between studies depending on the methods used. Vangl2 Ser517 was independently found to be phosphorylated by mass spectrometry analysis in different cell types [37, 38, 39], indicating physiological relevance. A recent study investigating the specificity of the Vangl PBM depending on phosphorylation of Vangl2 Ser517 (pSer517) revealed changes in its interactome [36], including changes in affinity towards PDZ domains 1, 2 and 3 of Scrib as well as Intu‐PDZ.

Thus, it is tempting to speculate that Fuzzy‐Inturned recruitment in PCP signalling is mediated via the interaction of Intu‐PDZ with Vangl‐PBM. However, PDZ domains occasionally also mediate protein‐lipid interactions [40, 41], and the N‐terminal portion of Intu containing the PDZ domain was reported to interact with phosphatidylinositol 3‐phosphate (PI(3)P) in protein‐lipid overlay assays [19].

We here describe the biochemical investigation of the lipid and protein binding properties of Intu‐PDZ. We do not observe membrane association of Intu‐PDZ in liposome sedimentation assays or an interaction with the core PCP protein Vangl2. However, we detect a phosphorylation‐dependent switch in the affinity of Vangl2 to Scribble PDZ domains. Finally, we determined the crystal structure of Intu‐PDZ, revealing a non‐canonical fold that rationalises our biochemical findings and suggests a molecular function divergent from classical PDZ domains.

## Materials and methods

### Protein purification

GST‐PDZ domain fusion constructs (Table S1) were cloned into the vector pCDF6P [42] and transformed into *E. coli* BL21(DE3) cells. Expression (16 h, 16 °C) was induced after 30‐min cold shock on ice by addition of 0.25 mm isopropyl‐β‐D‐thiogalactoside at an optical density of 0.8. Cells were lysed in lysis buffer (250 mm NaCl, 100 mm HEPES, 1 mm dithiothreitol (DTT), 1 mm MgCl_2_, pH 8) supplemented with protease inhibitor mix HP [Serva], 0.025 mg/mL DNase I and 1 mg/mL Lysozyme. Cleared lysates were incubated with glutathione agarose at 4 °C, the resin was washed with buffer (200 mm NaCl, 50 mm HEPES, 1 mm DTT, pH 8, at 4 °C) and proteins were eluted at 4 °C by addition of 20 mm glutathione and 10 mm DTT, or by PreScission cleavage overnight at 4 °C, respectively. Proteins were further purified by size exclusion chromatography (SEC70 10/300, [Bio‐Rad, Hercules, CA, USA] or HiLoad™ 16/600 Superdex75 pg, [Cytiva, Marlborough, MA, USA]) with SEC buffer (200 mm NaCl, 50 mm HEPES, 1 mm Tris(2‐carboxyethyl)phosphine (TCEP), pH 8) at 4 °C.

### Liposome cosedimentation assay

For generation of liposomes (Table S2), lipids were mixed in chloroform and dried by SpeedVac. Lipid films were resuspended in buffer L (25 mm HEPES, 250 mm NaCl, 1 mm MgCl_2_, pH 7.3) supplemented with 5% sucrose to a final lipid concentration of 2 mm. Multilamellar lipid vesicles were produced by five cycles of freezing in liquid nitrogen and thawing at 56 °C, and stored at −70 °C. Multilamellar vesicles were extruded 23 times through a polycarbonate membrane to obtain liposomes of 400 nm diameter before usage.

Proteins and liposomes were mixed in buffer L at final concentrations of 1 μm protein and 0.5 mm lipids, respectively. Reactions were incubated for 20 min at room temperature and liposomes pelleted at 20000× *g* for 20 min at 4 °C. Supernatant fractions were precipitated with acetone at −20 °C, and supernatant and pellet fractions were analysed by SDS/PAGE and Coomassie staining.

### GST‐pulldown

GST‐Intu‐PDZ and GST‐Scrib‐PDZ3 (Table S1) were expressed and purified essentially as described above but directly used for pull‐downs after immobilisation on GSH beads and washing. N‐terminally HA‐tagged Vangl2 cloned into pcDNA3 was expressed in HEK293T cells. Cells were lysed in CoIP buffer (40 mm HEPES, 120 mm NaCl, 10 mm MgCl_2_, 0.3% CHAPS, pH 7.4) supplemented with protease inhibitor mix HP [Serva] at 4 °C. 50 μL of GST‐PDZ slurry (1 : 1) was added to cleared HEK293T cell lysates, incubated for 1 h at 4 °C. GSH‐PDZ resins were pelleted (500× *g*, 5 min, 4 °C) and washed three times with 500 μL PBS (10 mm Na_2_HPO_4_, 1.8 mm KH_2_PO_4_, 137 mm NaCl, 2.7 mm KCl) on ice. Samples were analysed by SDS/PAGE and Coomassie staining or western blotting on polyvinylidene fluoride (PVDF) membranes using a semidry system. Membranes were blocked with TBS (20 mm Tris, 150 mm NaCl, pH 7.6) supplemented with 5% milk for 30 min, rinsed with TBS‐T (20 mm Tris, 150 mm NaCl, 0.05% Tween20, pH 7.6) and detection was performed with mouse α‐HA antibody [#16B12; BioLegend, San Diego, CA, USA] and α‐mouse‐HRP conjugate [#P0260; Dako/Agilent, Santa Clara, CA, USA] using ECL [Thermo Scientific, Darmstadt, Germany, SuperSignal West Pico PLUS] on a ChemoStar imager [Intas, Göttingen, Germany].

### Co‐immunoprecipitation

HEK293T cells (RRID:CVCL_0063; purchased authenticated from ATCC, Manassas, VA, USA) were transfected with the indicated plasmids (Table S1) using polyethylenimine. Cells were lysed in 800 μL CoIP buffer supplemented with protease inhibitor cocktail HP [serva]. The clear lysate was incubated with 20 μL M2 anti‐FLAG resin slurry (1 : 1 in Co‐IP buffer, [Sigma, Darmstadt, Germany]) for 3 h at 4 °C. Resin was pelleted (4000× *g*, 1 min, 4 °C), the supernatant removed, and the pellets washed three times with CoIP buffer on ice. Pellets were resuspended in 40 μL SDS‐loading dye and analysed by western blotting on PVDF membranes using a semi‐dry system. Membranes were blocked with TBS supplemented with 5% milk (30 min), washed with TBS‐T and detection performed with mouse α‐HA antibody [#16B12; BioLegend], or mouse α‐FLAG antibody [#F3165; Sigma], and α‐mouse‐HRP conjugate [#P0260; Dako] using ECL [Thermo Scientific, SuperSignal West Pico PLUS] on a ChemoStar imager [Intas].

### Biolayer interferometry

The synthetic peptides (0.5 mm) Vangl2‐PMD (^514^R‐L‐Q‐S‐E‐T‐S‐V^521^) and Vangl2‐pPMD (^514^R‐L‐Q‐pS‐E‐T‐S‐V^521^) [GenScript, Piscataway, NJ, USA] were biotinylated with biotin N‐hydroxysuccinimide [MedChemExpress, Monmouth Junction, NJ, USA] (2.5 mm) at 4 °C for 16 h. Samples were purified by HPLC [Agilent, Santa Clara, CA, USA, 1260 Infinity II HPLC system equipped with an autosampler and a DAD HS detector] on a Nucleodur C18 pyramid reverse‐phase column with a gradient of increasing acetonitrile concentration (0–50%) in PBS. Peak fractions were analysed via ESI‐MS and peptides lyophilised, followed by solubilisation at desired concentrations in PBS. Peptide–protein interactions were investigated using the Octet R2 instrument [Sartorius] and streptavidin conjugated biosensors [Sartorius, Göttingen, Germany] controlled by the build‐in software Octet® BLI Discovery 13.0.1.19. Sensor hydration was carried out in the final assay buffer (PBS, 0.05% Tween‐20) for 1000 s. The biolayer interferometry (BLI) assay was performed by loading the sensor with 100 nm biotinylated peptide for 120 s, association of PDZ domains (Table S1) at 20 μm concentrations for 300 s and dissociation in assay buffer for 300 s.

### X‐ray crystallography

Crystals were obtained in a sitting drop vapour diffusion experiment using the Morpheus Screen [Molecular Dimensions, Rotherham, UK] at 4 °C. 400 nL reservoir solution (condition A4: 37.5%(w/v) M1K3350, 0.1 m MB1, pH 6.5, 0.06 m divalents) were mixed with 400 nL protein at a concentration of 26 mg/mL. Crystals were mounted in nylon loops, cryo‐cooled in liquid nitrogen, and X‐ray diffraction data was collected at beamline 14.1, Helmholtz Zentrum Berlin [43]. The diffraction data showed high mosaicity but could be processed with the CCP4 software suit [44, 45, 46] using xia2 in DIALS [47]. Ccp4mg [48] was used to prepare an ensemble with MrBUMP [49] for molecular replacement in Phaser [50]. The model was built in iterative rounds of manual building in Coot [51] and refinement with Phenix (Table S3) [52].

## Results

### Intu‐PDZ does not bind lipid bilayers

Because the PDZ domain is a conserved feature of Inturned but unique to TLD family proteins, it likely mediates a specific function in the context of the Fuzzy‐Inturned complex. To investigate this question, we first followed up on a previous report that an N‐terminal fragment of Inturned containing the PDZ domain interacts with phosphatidylinositol‐3‐phosphate (PI(3)P) in protein‐lipid overlay assays [19]. Although PDZ domains are generally considered protein–protein interaction domains, some representatives show membrane binding, also to phosphoinositides [40, 41]. We therefore tested a putative Intu‐PDZ interaction with lipid bilayers by liposome cosedimentation assays. We used liposomes of different membrane compositions, including a neutral palmitoyl‐oleoyl (PO) mix, dioleoyl (DO) liposomes with high packing defects, PI(3)P‐rich (2 mol%) liposomes or charged liposomes with phosphatidylinositol‐3,5‐bisphosphate (1 mol% PI(3,5)P_2_) and phosphatidylserine (2 mol% PS) (Table S2). In addition to testing the human and *Drosophila melanogaster* Intu‐PDZ domains, we used HSV2 (from *Kluyveromyces lactis*) that was shown to interact with phosphoinositides as a positive control [53]. While HSV2 was recruited to phosphoinositide containing membranes as expected, we could not observe recruitment of Intu‐PDZ domains to PI(3)P‐containing liposomes or other membrane compositions (Fig. 1).

**Fig. 1:**
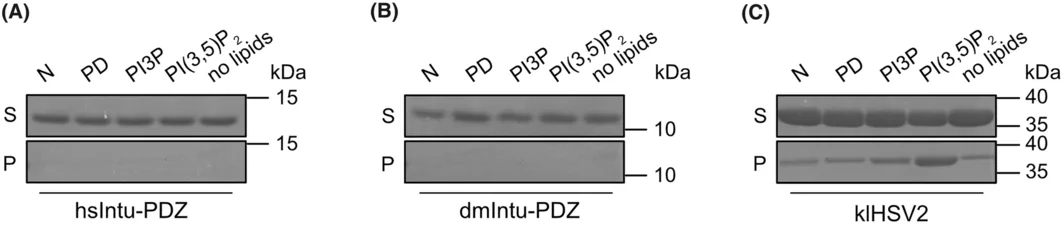
Liposome binding studies with Intu‐PDZ. Co‐sedimentation assays of (A) human (hs) and (B) *Drosophila melanogaster* (dm) Intu‐PDZ with different liposome compositions (representative of three independent repeats). (C) *Klyveromyces lactis* (kl) HSV2 serves as a positive control. PO: palmitoyl‐oleoyl, DO: dioleoyl, PI(3)P: phosphatidylinositol‐3‐phosphate (2 mol%), PI(3,5)P2: phosphatidylinositol‐3,5‐bisphosphate (1 mol%), PS: phosphatidylserine (2 mol%).

### PDZ interactions with the Vangl2‐PBM

Several lines of evidence suggest a close functional relationship between Vangl and planar polarity effector proteins. The proteins localise to the proximal side of wing cells in *Drosophila*, and while lack of Inturned does not alter Vang localisation, it is epistatic to the PCP core protein, which could be explained by a direct interaction [54, 55, 56].

Thus, because Intu and Vangl are functionally linked, we next investigated a potential interaction between Intu‐PDZ and Vangl. Because the PBMs of Vangl1 and Vangl2 are identical (Fig. S1) [57], we focus on Vangl2 in the following. We used co‐immunoprecipitation and GST pulldown assays with Vangl2 and Intu‐PDZ to test for direct binding. Scrib‐PDZ3, a well‐established binding partner of Vangl2, served as a positive control. Both assays showed an interaction between Scrib‐PDZ3 and Vangl2, but not between Intu‐PDZ and Vangl2 (Fig. 2). These data argue against a direct recruitment of Fuzzy‐Inturned to the proximal cell membrane via Vangl2‐PBM.

**Fig. 2:**
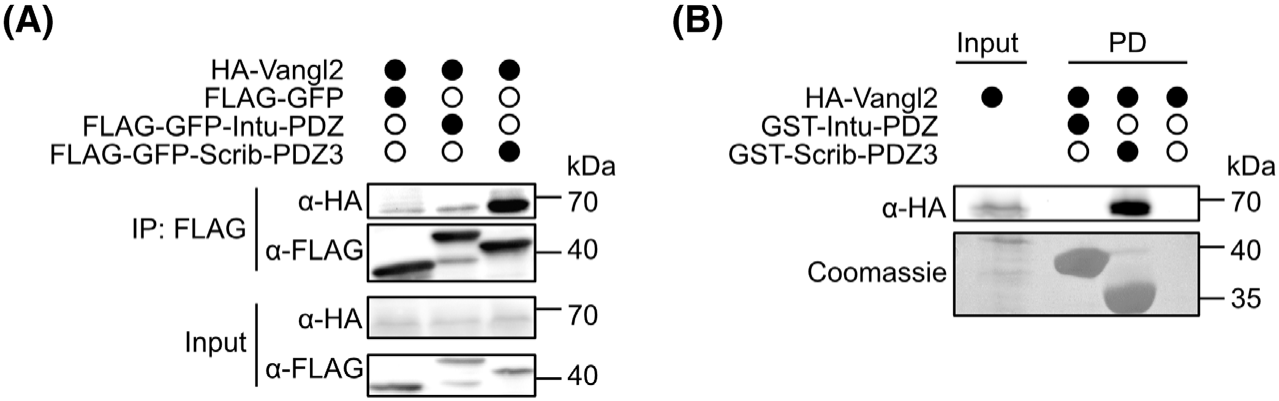
Intu‐PDZ does not bind the canonical PDZ binding motif of Vangl2. (A) GST pulldown assay (representative of three independent repeats) with Intu‐PDZ and Scrib‐PDZ3 as bait proteins and HA‐Vangl2 expressed in HEK293 cell lysate as prey. (B) Co‐immunoprecipitation of Vangl2 with FLAG‐GFP tagged PDZ domains of Inturned and Scribble (representative of three independent repeats). FLAG‐GFP was used as a negative control.

We asked whether the interaction between Intu and Vangl may depend on a post‐translational modification. Recently, Montserrat‐Gomez and colleagues investigated the interactome of Vangl2 PDZ binding motifs in a high‐throughput holdup assay [36]. An interaction between Vangl2 PBM phosphorylated at Ser517 and Intu‐PDZ was detected. By contrast, the unphosphorylated peptide did not yield an interaction. Interestingly, this phosphorylation also increased the affinity of Vangl2 towards Scrib‐PDZ2 but diminished it towards Scrib‐PDZ1 and Scrib‐PDZ3.

To validate whether phosphorylation of Vangl2 modulates its affinity for PDZ domains, we purified Intu‐PDZ as well as all four PDZ domains of Scribble (Fig. S2A) and employed biolayer interferometry (BLI). We investigated binding of the eight amino acid long C‐terminal peptide of Vangl2 comprising the PBM in its unphosphorylated form (wt) or phosphorylated at Ser517 (pS517) (Fig. 3A–E).

**Fig. 3:**
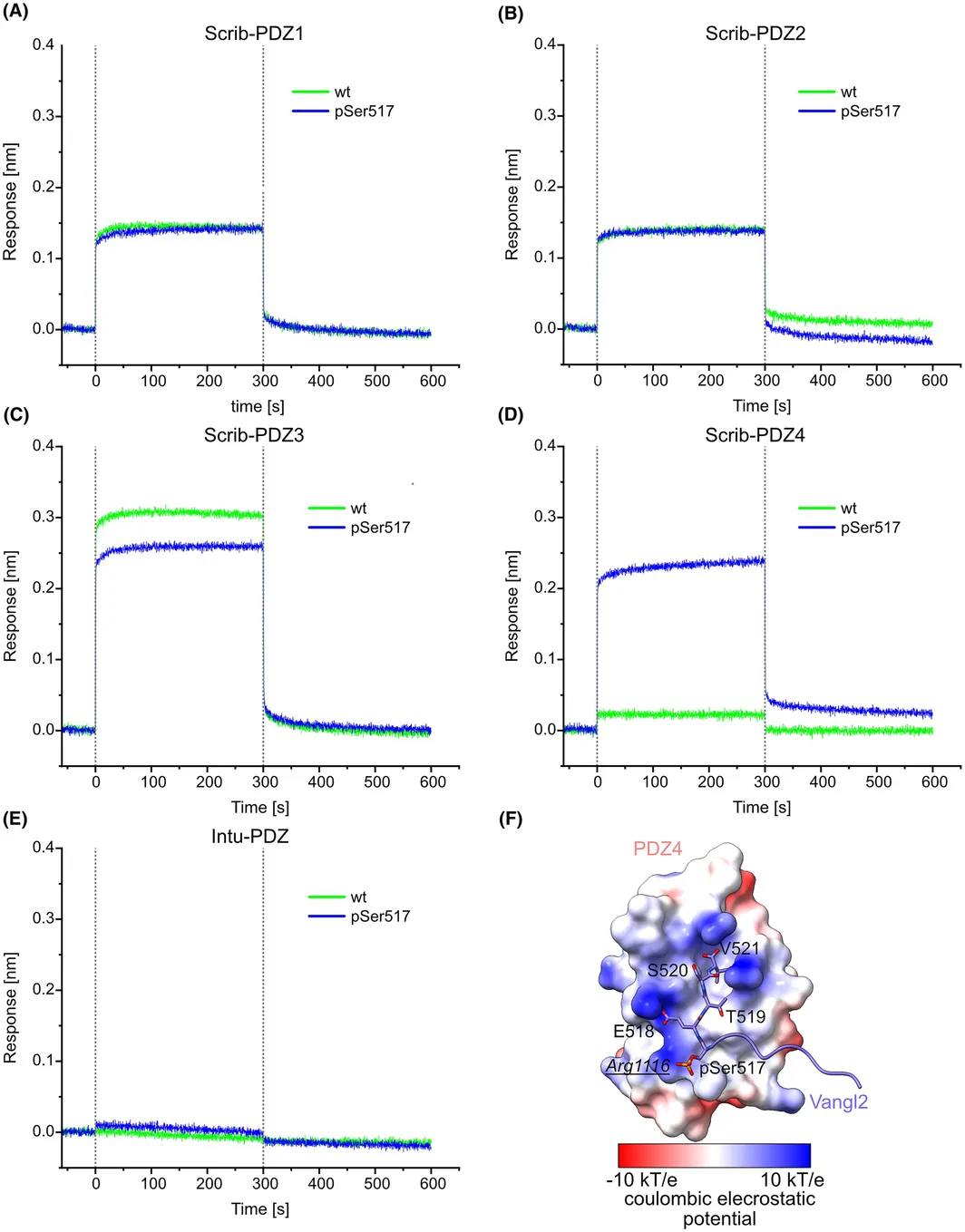
Phosphorylation dependent interactions of Vangl PBM with PDZ domains of Intu and Scrib. Biolayer interferometry measurements of Vangl PBM either phosphorylated (pSer517) or unphosphorylated (wt) at Ser517 with (A) Scrib‐PDZ1, (B) Scrib‐PDZ2, (C) Scrib‐PDZ3. (D) Scrib‐PDZ4. (E) Intu‐PDZ. Representative examples of two experiments. (F) AlphaFold 3 prediction of Scirb‐PDZ4 with pSer517 Vangl2. Sequence labels are shown for the PBM and Arg1116 of Scribble (underlined, italics).

In accordance with previously published data, we observe interactions between unphosphorylated Vangl2 peptide with Scrib‐PDZ1‐3 (Fig. 3A–C), but neither with Scrib‐PDZ4 nor Intu‐PDZ (Fig. 3D,E) [39, 40]. Upon phosphorylation of Ser517, Scrib‐PDZ1 and ‐PDZ2 interaction with Vangl2‐PBM was largely unaffected, but weaker binding of Scrib‐PDZ3 and a gain of binding of Scrib‐PDZ4 can be observed (Fig. 3D). However, Intu‐PDZ also did not bind phosphorylated Vangl2‐PBM (Fig. 3E).

AlphaFold‐3 predictions [58] provide a plausible explanation for the phosphorylation‐specific binding of Scrib‐PDZ4 to Vangl2‐PBM. The model of Scrib‐PDZ4 with the unphosphorylated PBM reaches an ipTM of 0.54, hence indicating an unlikely complex, while the phosphorylated Vangl2 Scrib‐PDZ4 complex yields an ipTM of 0.78 (Fig. 3F and Fig. S2B–F). The predicted binding mode of Vangl2 and Scrl‐PDZ4 was independent of whether one or three copies were used as input (Fig. S2B,D). The interaction can thus also occur with Vangl2 trimers, which represent the likely assembly of the protein [25, 26]. The predicted model shows the phosphorylated serine in close proximity to Arg1116 of Scribble, indicating a potential electrostatic interaction. This might contribute additional binding energy required for stable binding, which is not possible with unphosphorylated peptide. The AlphaFold prediction thus provides a mechanistic explanation for the phosphorylation‐specific interaction. In contrast, residues of Scirb‐PDZ1, ‐PDZ2 and Scirb‐PDZ3 at equivalent positions are small and nonpolar (Ala or Val) (Fig. S3), explaining why they are largely indifferent regarding phosphorylation at Vangl2 Ser517.

### Crystal structure of Intu‐PDZ

The uncharacteristic behaviour of Intu‐PDZ in the interaction studies prompted us to more closely investigate its structure. Intu‐PDZ is uncommonly large in relation to other PDZ domains—107 amino acids compared to conventionally 80–90 (Fig. S3) [22, 57, 59, 60]. The AlphaFold prediction [61] indicates the presence of two additional helices; however, modelled with a low confidence score. To obtain further structural insight, we determined the crystal structure of the human Intu‐PDZ domain (Fig. S4) at 2.5 Å resolution (Table S3). To our surprise, initial molecular replacement attempts with an AlphaFold2 prediction [62] failed. A solution was only found after preparation of a molecular replacement model from an ensemble with MrBump and ccp4mg [48, 49] (Fig. 4A). Structural comparison with the canonical third PDZ domain of Scribble (Scrib‐PDZ3) shows that two additional helices are placed in the canonical binding groove of PDZ binding motifs, like in the case of Scribble, the conserved C‐terminal peptide of Vangl1 and Vangl2 (Fig. 4B,C).

**Fig. 4:**
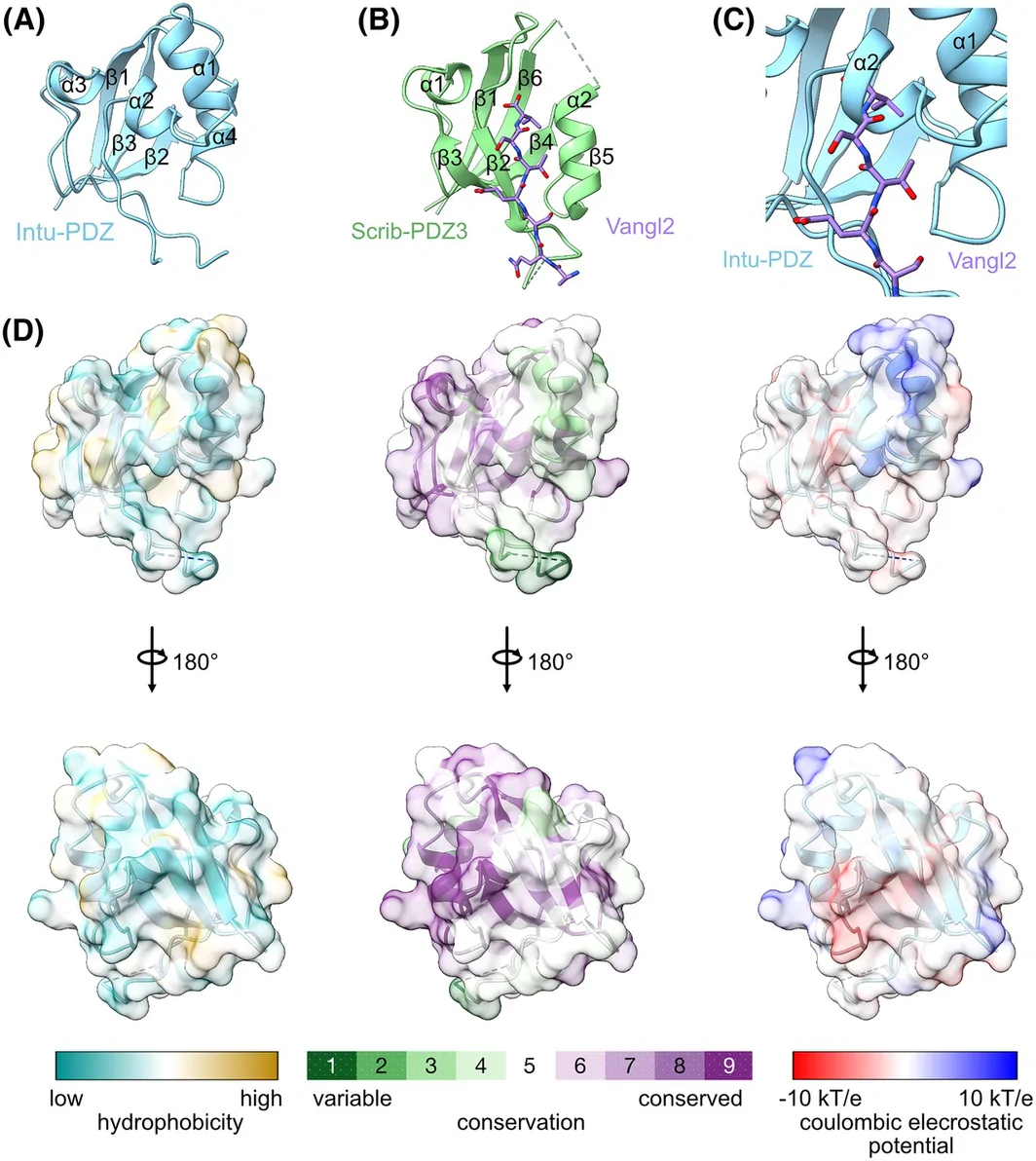
Intu‐PDZ is a non‐canonical PDZ‐like domain. (A) Structure of Intu‐PDZ shown in cartoon representation with secondary structure elements labelled consecutively. (B) Structure of the canonical third PDZ domain of Scribble bound to the C‐terminal Vangl2 PDZ binding motif. PDB ID: 6XA6 [35]. Secondary structure elements are labelled consecutively. (C) Superposition of (A) and (B) with focus on the putative binding groove shows the Vangl2 peptide clashing with one of the additional helices (α2) found in Intu‐PDZ; Scrib‐PDZ3 hidden. (D) Surface representation of Intu‐PDZ in two views, coloured by hydrophobicity, conservation and coulombic electrostatic potential.

Search of the protein data bank [63] with DALI [64] did not yield any structures with similar architecture as Intu‐PDZ. This domain with the additional helices α1 and α2 blocking the PDZ motif binding site thus apparently represents a novel PDZ‐like fold, which may also explain the local inaccuracy of the AlphaFold prediction (Fig. S5) [61]. Helix α2 is conserved [65] in Inturned across species, suggesting that the unconventional fold relates to its function (Fig. 4D). Interestingly, the surface patch with the highest conservation [65] is found on the side opposite the canonical PBM binding groove. The patch shows an even charge distribution [66] and might represent a protein–protein interaction interface (Fig. 4D). However, there is no basic or hydrophobic surface region observable that could represent a membrane binding site.

Thus, the crystal structure provides a molecular explanation why we could not confirm the previously suggested interactions of Intu‐PDZ with membranes or (phospho‐)PDZ binding motifs.

## Discussion

Taken together, our data show that Intu‐PDZ does not represent a canonical PDZ domain. Structurally, the domain represents a novel PDZ‐like fold that so far is unique in the protein data bank [64]. The defining feature is the insertion of two additional helices in the classical PDZ fold that block the interaction site for PDZ binding motifs. Functionally, Intu‐PDZ therefore does not bind PBM‐containing proteins such as Vangl2, but its surface properties make a role in mediating protein–protein interactions beyond PDZ binding motifs conceivable. Identifying the binding partner(s) of Intu‐PDZ remains an unresolved challenge.

Thus, Vang/Vangl1/2 do not recruit PPE/CPLANE complexes to the proximal side of the cell via Intu‐PDZ. We can only speculate at this point which other mechanisms may be involved. Localisation of Fuzzy‐Inturned to the ciliary base involves the BAR domain adaptor protein ciBAR1 [11, 17], which could also play a role in PCP. Alternatively, Prickle and Flamingo/Celsr may represent receptors for Fuzzy‐Inturned complexes at the proximal membrane.

Interestingly, we observe a phosphorylation‐dependent switch in the binding of different PDZ domains of Scribble to Vangl2. Phosphorylation of both PDZ domains and their binding motifs is a common theme in the regulation of PDZ‐mediated interactions [67, 68, 69, 70, 71, 72, 73, 74, 75, 76, 77, 78, 79, 80]. Scribble PDZ1‐3 bind both phosphorylated and unphosphorylated Vangl, but phosphorylation of Vangl2 increased Scrib‐PDZ4 binding. Structure modelling suggests that an additional salt bridge between the phosphate group and an arginine unique to PDZ4 is required to stabilise the interaction. This may explain previous conflicting studies that reported an interaction of Vangl with PDZ domains 2, 3 and 4 by pulldown experiments, while isothermal titration calorimetry measurements showed interactions with PDZ domains 1, 2, and 3, but not 4 [34, 35]. The discrepancy between our results on phospho‐specificity and the findings of a high‐throughput study [36] may result from differences in the experimental setup.

It was also shown that phosphorylation of the PBM abolishes affinity between Scribble and Mutated in Colorectal Cancer (MCC) [81]. Phosphorylation of Vangl proteins and the *Drosophila* ortholog Vang was reported at multiple sites and is well established as a regulating factor. Vang dephosphorylation by the protein phosphatase 1 regulates its subcellular localisation [82] and Wnt‐induced Vangl phosphorylation has been described to be required for establishment of planar polarity [83].

Disentangling the phosphorylation at distinct sites of Vang and how these regulate the interactome remains to be resolved. This is especially true for the combination of PDZ domains of Scribble. Vangl was recently shown to form a trimeric complex *in vitro* [25, 26]. How four PDZ domains bind this trimer—and if cooperatively, competitively, or forming higher order networks—remains to be resolved. It is noteworthy that allostery plays a role in PDZ domain mediated interactions [84, 85] with adjacent domains altering PDZ affinity to interaction partners [86, 87], which could add an additional layer of complexity. Potentially, Scribble and Vangl2 can form two functionally distinct complexes depending on the PBM phosphorylation state.

## Author contributions

SW was involved in conceptualisation, investigation, writing—original draft, review & editing, formal analysis. JB performed investigation and formal analysis. CG and LM performed investigation. DK was involved in conceptualisation, writing—review & editing, formal analysis, funding acquisition.

## Supporting information


**Table S1** List of plasmids used in this study.
**Table S2** Composition of liposomes used in this study.
**Table S3** Data collection and refinement statistics of Intu‐PDZ.
**Fig. S1**. Sequence alignment of human Vangl1 and Vangl2.
**Fig. S2**. Scrib‐PDZ4 interacts with VanglL‐2 PBM.
**Fig. S3**. Sequence alignment of Intu and Scrib PDZ domains.
**Fig. S4**. Purification of Intu‐PDZ.
**Fig. S5**. Comparison of the Intu‐PDZ crystal structure and the prediction from the AlphaFold Database.

## Data Availability

The structural data that support these findings are openly available in the wwPDB [63] at https://doi.org/10.2210/pdb9T1R/pdb.
