# Supplementary material for: The planar cell polarity protein Vangl2 interacts with the PDZ‐domains of Scribble but not with a unique PDZ‐like domain in Inturned
Source: FEBS Lett. 2026 Mar 8;600(18):2765–76. doi: 10.1002/1873-3468.70319 (PMC13618291; doi:10.1002/1873-3468.70319)
Supplement: Supplementary file 1 [file FEB2-600-2765-s001.pdf]

Supporting Information

The planar cell polarity protein Vangl2 interacts with the PDZ-domains of Scribble but not with a unique PDZ-like domain in Inturned

Stephan Wilmes*, Jan Brysch, Carmen Gelze, Lilli Meier, Daniel Kümmel*

Table S1 List of plasmids used in this study.

Protein	Uniprot ID	amino acids	vector	stock #	host
KlHSV2	Q6CN23	1-339	pET28HS	1621	BL21
HsIntu-PDZ	Q9ULD6	147-268	pCDF6P	1433	BL21
DmIntu-PDZ	Q9VPH0	122-217	pCDF6P	1317	BL21
HsScrib-PDZ1	Q14160	700-816	pCDF6P	2242	BL21
HsScrib-PDZ2	Q14160	860-950	pCDF6P	2243	BL21
HsScrib-PDZ3	Q14160	1002-1092	pCDF6P	1628	BL21
HsScrib-PDZ4	Q14160	1100-1194	pCDF6P	2291	BL21
HsVangl2	Q9ULK5	1-521	pCDNA3-HA	1618	HEK293T
HsIntu-PDZ	Q9ULD6	147-268	pCDNA3-FG	1620	HEK293T
HsScrib-PDZ3	Q14160	1002-1092	pCDNA3-FG	1629	HEK293T
FLAG-GFP			pCDNA3-FG	1710	HEK293T

Table S2 Composition of liposomes used in this study.

PO: palmitoyl-oleoyl, DO: dioleoyl, PI(3)P: phosphatidylinositol-3-phosphate, PI(3,5)P_2: phosphatidylinositol-3,5-bisphosphate, PS: phosphatidylserine, PE: phosphatidylethanolamine, PC: phosphatidylcholine.

Lipid mix	Composition
PO	18 mol% POPE, 82 mol% POPC
DO	18 mol% DOPE, 82 mol% DOPC
PO+PI(3)P	16 mol% POPE, 82 mol% POPC, 2 mol% PI3P
PO+PI(3,5)P_2/PS	15 mol% POPE, 82 mol% POPC, 2 mol% PS, 1 mol% PI(3,5)P_2

Table S3 Data collection and refinement statistics of Intu-PDZ.

PDB ID	9T1R
Space group	P 2_1
Wavelength (Å)	0.918
Unit cell	
a (Å)	38.15
b (Å)	43.59
c (Å)	60.36
α (°)	90.0
β (°)	94.6
γ (°)	90.0
Resolution (Å)	60.16-2.10 (2.16-2.10)
Unique Reflections	10933 (902)
Multiplicity	6.20 (6.40)
I/σ	10.60 (3.10)
Completeness (%)	93.90 (96.40)
R_{meas} (%)	10 (50)
CC(1/2)	1.00 (0.95)
Wilson B factor (Å)	21.04
**Refinement**	
Resolution (Å)	35.3-2.50
Reflections	6493
R_{work} (%)	27.7
R_{free} (%)	32.7
R.M.S.D bond length (Å)	0.004
R.M.S.D. bond angles (°)	0.974
Ramachandran	
favoured (%)	98.42
allowed (%)	1.58
outlier (%)	0.0
B value (Å^2)	
Protein	22.84
Waters	22.51

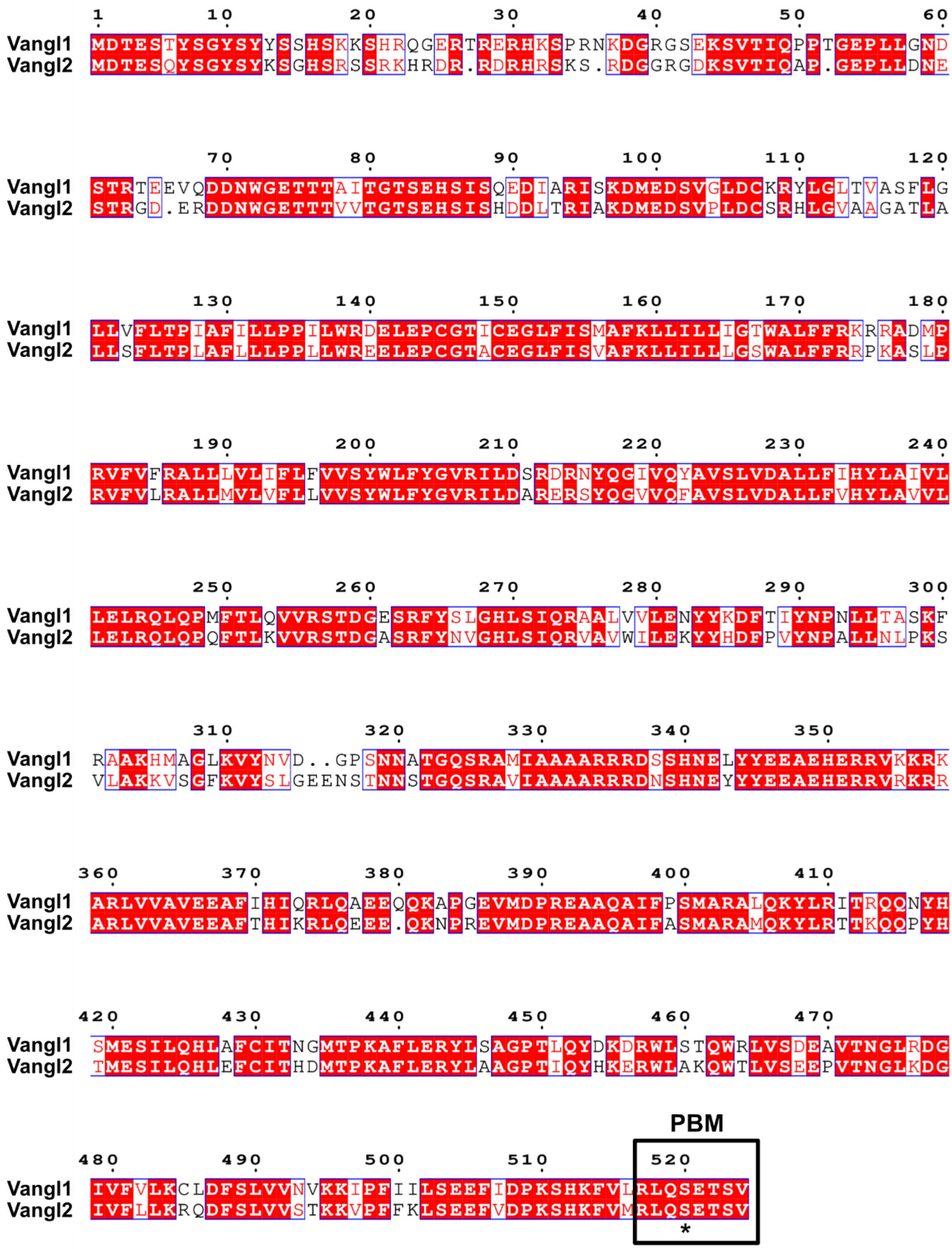

Fig. S1: Sequence alignment of human Vangl1 (UniProt ID: Q8TAA9) and Vangl2 (UniProt ID Q9ULK5). Sequence numbering refers to Vangl1. The C-terminal PDZ binding motifs (PBM) and discussed phosphorylated Serin residues (Ser517 in Vangl2 equivalent to Ser520 in Vangl1) are highlighted by a box and asterisk, respectively. Alignment was generated wit Clustal Omega [57].

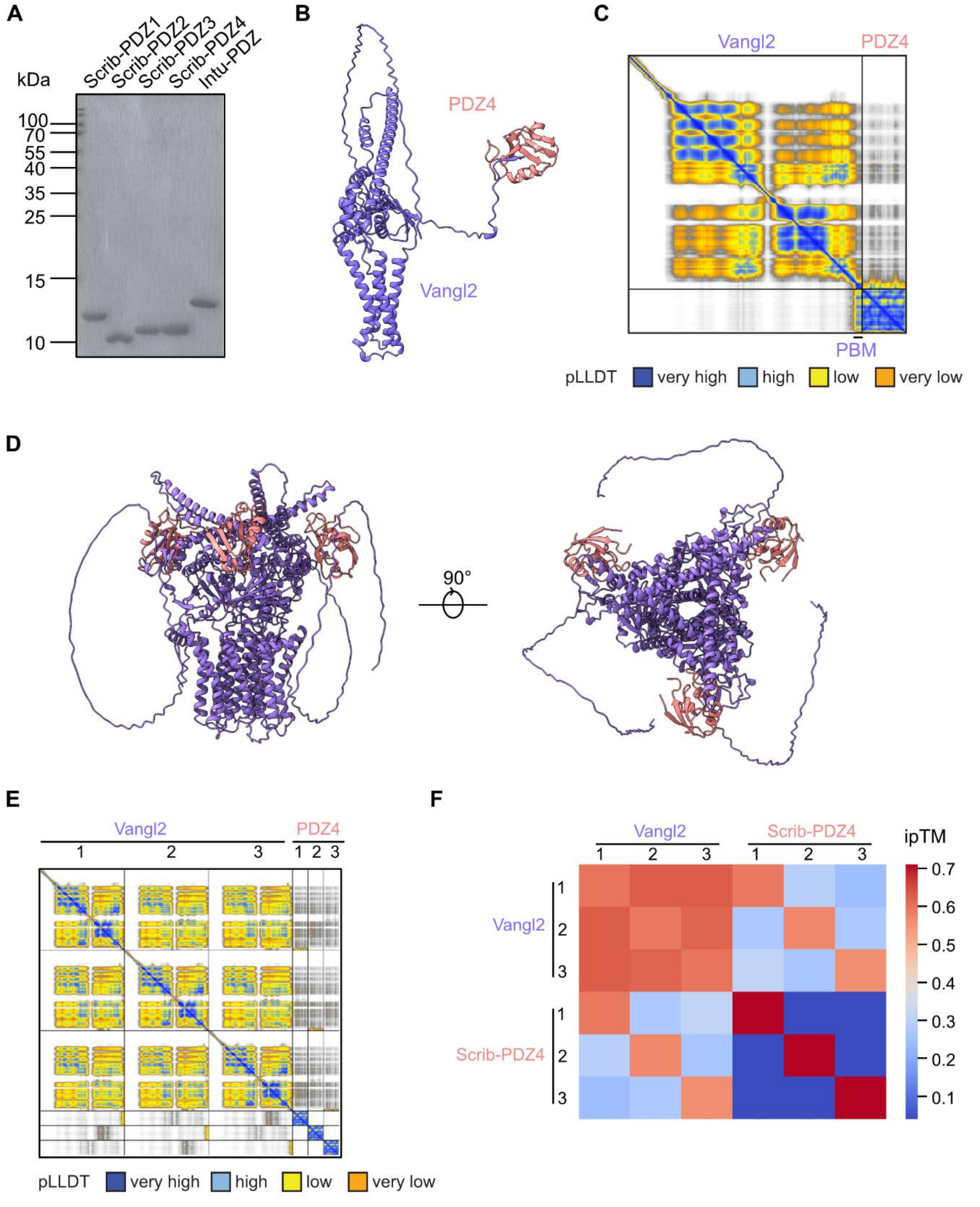

Fig. S2: Scrib-PDZ4 interacts with Vangl-2 PBM. **(A)** SDS-PAGE analysis of purified PDZ domains used for BLI measurements. **(B)** AlphaFold3 prediction of Vangl2 pSer517 in complex with Scrib-PDZ4 with ipTM of 0.78 and pTM of 0.46 in cartoon representation colored by chain. **(C)** Predicted algined error (PAE) plot for model from (B) colored by predicted local distance difference test (pLDDT) scores. **(D)** AlphaFold3 prediction of three Vangl2 pSer517 protomers in complex with three Scrib-PDZ4 protomers with ipTM of 0.61 and pTM of 0.63. **(E)** PAE plot for model from (D) colored by pLDDT scores. **(F)** Pairwise ipTM score heatmap for prediction (D) prepared using Ash100. AF3 Results Visualization Notebook. GitHub (2024); https://github.com/Ash100/Biopython/blob/main/AF3_Results_Visualization.ipynb (accessed 06.01.2026).

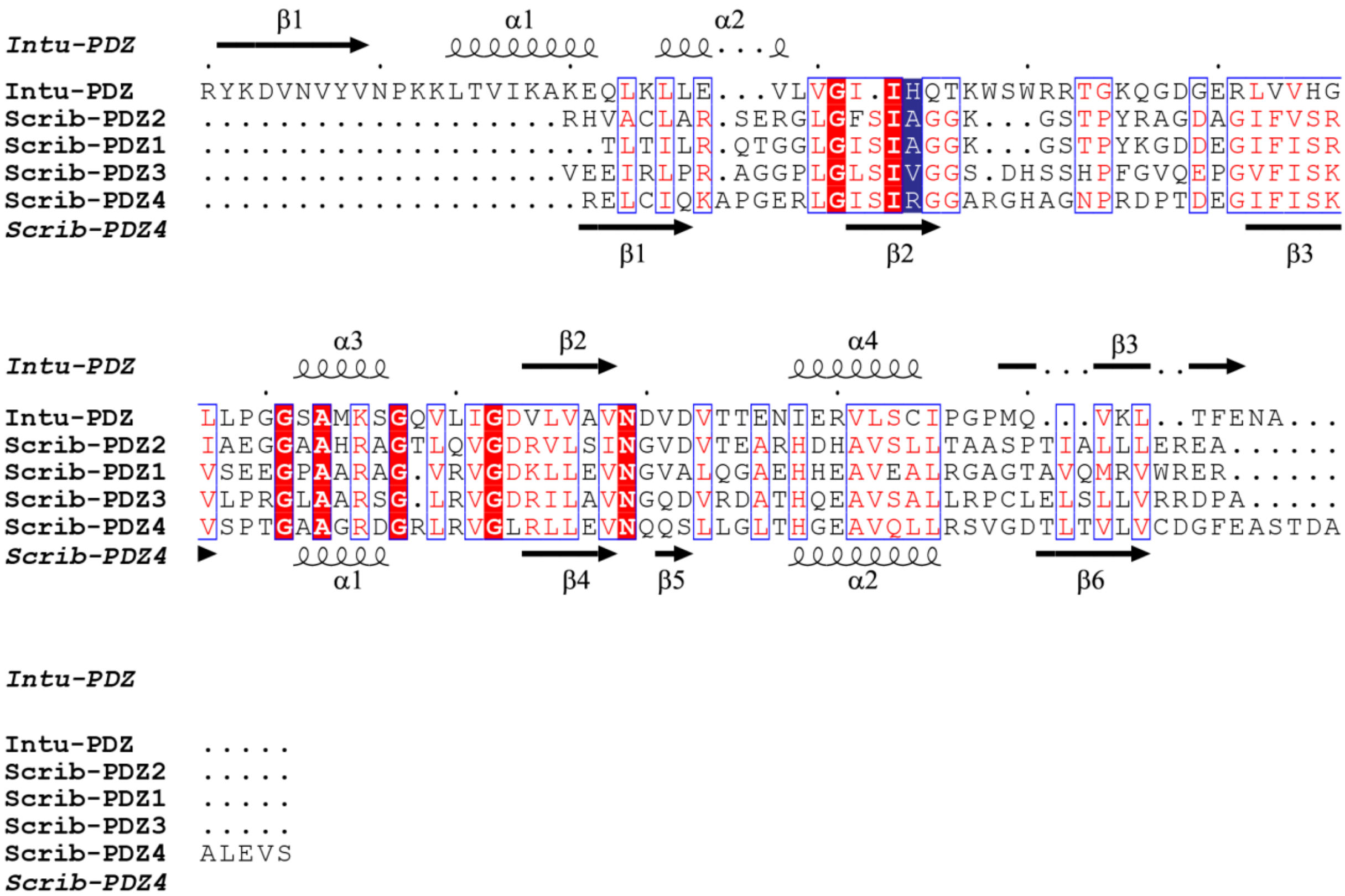

Fig. S3 Sequence alignment of Intu (UniProt ID Q9ULD6) and Scrib (UniProt ID Q14160) PDZ domains [57]. Secondary structure elements were inferred from crystal structures (Scrib-PDZ3 PDB ID: 6EEY [59]). Scrib-PDZ4 Arg1116 is highlighted by a blue background. The annotation of secondary structure elements was prepared with ENDscript - https://endscript.ibcp.fr [60].

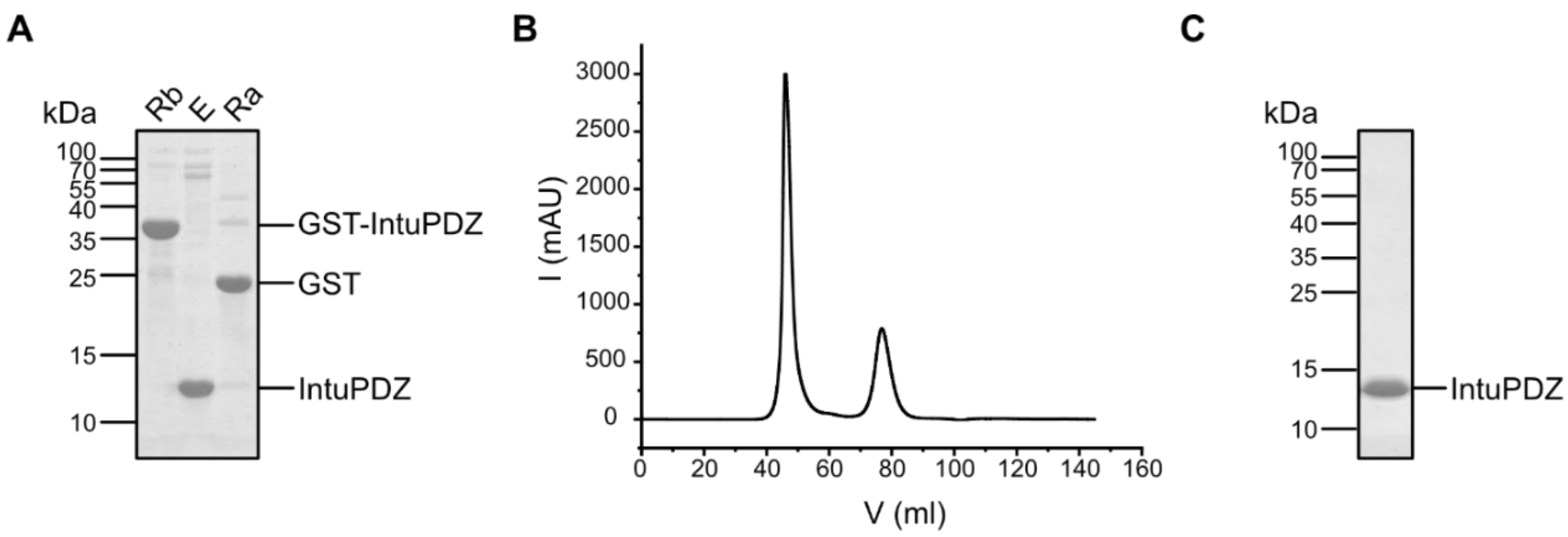

Fig. S4 Purification of Intu-PDZ. **(A)** SDS-PAGE gel of GSH-affinity purification; Rb - resin before PreScission protease cleavage, E - elution fraction, Ra - resin after elution. **(B)** Size exclusion chromatogram on HiLoad Superdex 75 pg 16/600 column. **(C)** SDS-PAGE analysis of the size excluison peak fraction (79 ml) in (B).

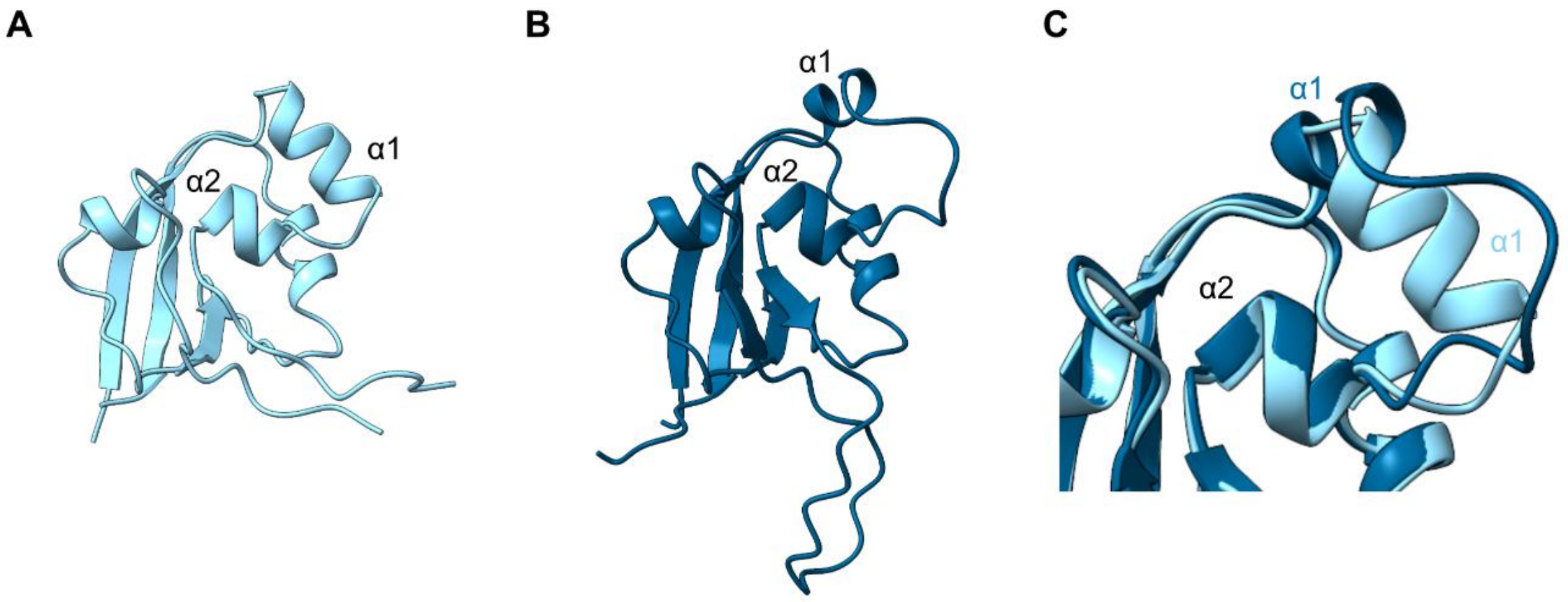

Fig. S5: Comparison of the Intu-PDZ crystal structure and the prediction from the AlphaFold Database [61]. **(A)** Crystal structure of Intu-PDZ. **(B)** Cropped AlphaFold database prediction of Inturned (AF-Q9ULD6-F1-v6) showing the PDZ domain. **(C)** Close-up of the superposition of the Intu-PDZ crystal structure (cyan) and AlphaFold prediction (dark blue).
